# Determinants of gestational diabetes mellitus: A case control study in a district tertiary care hospital in south India

**DOI:** 10.4103/0973-3930.62599

**Published:** 2010

**Authors:** Mamta Bhat, Ramesha K. N., Sankara P. Sarma, Sangeetha Menon, Sowmini C. V., Ganesh Kumar S.

**Affiliations:** 1 Department of Obstetrics and Gynecology, Sree Chitra Tirunal Institute for Medical Sciences, Thiruvananthapuram, India; 4 Department of HRRC/ICMR/ Medical College, Sree Chitra Tirunal Institute for Medical Sciences, Thiruvananthapuram, India; 2 Department of Neurology, Sree Chitra Tirunal Institute for Medical Sciences, Thiruvananthapuram, India; 3 Department of Biostatistics Sree Chitra Tirunal Institute for Medical Sciences, Thiruvananthapuram, India; 5 Department of Preventive and Social Medicine, JIPMER, Puducherry, India

**Keywords:** Gestational Diabetes Mellitus, Determinants, Chi square test, Odds Ratio

## Abstract

**Objective:**

To study the determinants of Gestational Diabetes Mellitus (GDM).

**Design:**

Case-control study. Setting: Sri Avittom Thirunal Hospital, Thiruvananthapuram district, Kerala, South India.

**Participants:**

300 GDM women as cases and 300 age-matched controls.

**Study variables:**

Sociodemographic characteristics, pre-pregnancy Body Mass Index (BMI), menstrual history, obstetric history, infertility history, family history of diabetes in first degree relatives, recurrent urinary tract infection (UTI), and moniliasis.

**Statistical analysis:**

T-test, Fishers Exact Test, Chi square test, Adjusted Odds Ratio with 95% CI. Results: Pre-pregnancy BMI ≥ 25 (*P* < 0.001, OR = 2.7), irregular menstrual cycle (*P* = 0.006), treatment for infertility (*P* = 0.001, OR = 3.3), family history of diabetes (*P* = 0.001, OR = 4.5), history of diabetes in mother (*P* = 0.003), previous pregnancy losses (*P* = 0.04), past GDM (*P* = 0.035), prematurity (*P* = 0.01), pre-eclampsia (*P* = 0.04), polyhydramnios (*P* < 0.001, OR = 6.0), UTI (*P* < 0.001, OR = 3.2), and moniliasis (*P* < 0.001, OR = 7.6) were significantly associated with present GDM.

**Conclusion:**

Early identification of women at risk of GDM and prompt treatment is recommended to prevent complications.

## Introduction

Gestational diabetes mellitus (GDM), which is defined as the onset or recognition of glucose intolerance during pregnancy, is associated with an increased risk of perinatal morbidity and mortality. Women diagnosed with GDM are at an increased risk of developing diabetes in the future. The prevalence of GDM is high in the Indian population as compared to other populations of Southeast Asia.[1] In south India, the prevalence of GDM has increased from 1% in 1998[2] to 16.55% in 2004.[1] Gestational diabetes is a condition that can be effectively controlled, thereby decreasing the associated risks and eventually leading to the delivery of healthy infants.

The factors that have been postulated to influence the risk of GDM among the mothers include obesity, positive family history of diabetes in first-degree relative, treatment for infertility, polyhydramnios, recurrent UTI, recurrent moniliasis, history of still birth, delivery of a large infant (> 4 kg), unexplained neonatal death, prematurity, pre-eclampsia in multipara, diabetes in previous pregnancy, and advancing maternal age. In developing countries, evidence with regard to the association between these factors and GDM is scarce. Those that have been conducted have often had an inadequate control and lack of statistical power, resulting in inconclusive evidence for determinants of GDM in developing countries. In this context a case-control study was conducted to elucidate some of the major risk factors for GDM.

## Materials and Methods

This case-control study was carried out from August 2007 to June 2008 at Sri Avittom Thirunal Hospital, Thiruvananthapuram district, Kerala, South India. This is a tertiary care hospital and its maternity service is a referral center for the care of high-risk, pregnant women throughout this and the neighboring districts.

Patients were monitored with Glucose Challenge Test (GCT) at 24 – 28 weeks and 32 – 34 weeks, or whenever any risk factor developed during pregnancy. If the GCT was positive, a gestational diabetes status was confirmed with the Oral Glucose Tolerance Test (OGTT). Patients with high risk of developing GDM were screened with OGTT on their first antenatal visit. Thus these patients who developed glucose intolerance were included in the study group. The control group included the next woman of the same age, who had a normal GCT at 24 – 28 weeks, followed by a normal OGTT with 100 gm of glucose (age-matched control). The prenatal patients were given a 50 gm GCT and if the plasma glucose value after one hour exceeded 130 mg/dl, a 100 gm OGTT was performed after overnight fasting. Plasma samples were then drawn at one, two, and three hours after administration of glucose. For the purpose of this study, the OGTT results were interpreted by the National Diabetes Data group values. Accordingly the abnormal values were defined as follows: FBS - > 105 mg%, one hour - > 190 mg%, two hours - > 165 mg%, and three hours - > 145 mg%. If two or more values were abnormal, the patient was classified as a gestational diabetic. The exclusion criteria included women with a diagnosis of diabetes prior to pregnancy.

The risk factors that were assessed included sociodemographic characteristics, menstrual history, obstetric history (h/o previous pregnancy losses, macrosomia, congenital anomalies, prematurity, diabetes in previous pregnancy, pre-eclampsia, polyhydramnios), history of infertility, family history of diabetes in first-degree relatives, recurrent UTI, moniliasis, and premature labor pains. On examination, a note was made on the height and weight. Specific question regarding pre-pregnancy weight, that is, the weight prior to pregnancy or that recorded in the first prenatal visit in early pregnancy was noted for calculating the pre-pregnancy BMI (kg/m^2^). A complete general examination was carried out including heart rate and blood pressure. On abdominal examination, symphysio-fundal height was measured, and macrosomia or increased liquor, if detected clinically, and confirmed with ultrasound (effective fetal weight > 4 kg; amniotic fluid index > 25) was noted. A nuclear family consists of single married couple and their children, while a joint family consists of married couples and their children who live together in the same household. In three generation family, there are representatives of three generations.

For an alpha error of 5%, for a power of 80%, assuming the prevalence of gestational diabetes in India was 16.55%, and the odds ratio, 2, the minimum sample size was estimated to be 215 each of cases and controls. The data was analyzed by the use of SPSS version 12. A t-Test was performed to compare these variables. A Chi square test and Odd's ratio (Crude and adjusted) were calculated. A *P* value of < 0.05 was considered to be statistically significant. As GDM was a multifactorial condition, we used multiple logistic regression analysis to assess their independent effects of each variable. Adjusted odds ratios and 95% confidence intervals were calculated from the logistic regression analysis.

## Results

During the study period from August 2007 to June 2008, all the 338 cases of diabetes complicating pregnancy, who attended the clinic, were included. Of these patients, 38 were excluded as they had diabetes prior to pregnancy. The remaining 300 patients with GDM were included as cases and compared with 300 age-matched controls. The mean age of cases was 26.63 (± SD = 4.547) and the mean age of controls was 26.43 (± SD = 4.412). The t-test done showed no significant difference between the two (t = −0.4: df = 298; *P* = 0.7). 60.7% (n = 182) of the cases were ≥ 25 years, while 39.3% (n = 118) were < 25 years.

Around three quarters of the cases and controls were Hindus, half of them were from rural areas and studied high school level (eighth to tenth standard) education and had no difference in monthly family income. The number of primigravidae was almost equal to the number of multigravidae in the study group. The difference seen between cases and controls among different occupation groups was found to be significant (χ^2^ = 8.12, *P* = 0.02) [Table 1].

**Table 1:** Baseline characteristics

Determinants	Cases (%)	Controls (%)	χ^2^, *P*
Religion			
Hindu	210 (75.3)	242 (80.7)	4.69, 0.096
Muslim	40 (11.3)	28 (9.3)	
Christian	50 (16.7)	30 (10.0)	
Residence			
Rural	160 (53.3)	144 (48.0)	3.16, 0.205
Semi-Urban	96 (32.0)	88 (29.3)	
Urban	44 (14.7)	68 (22.7)	
Education			
Illiterate	0 (0)	2 (0.7)	9.11, 0.167 (df = 6)
Primary(first to fourth)	0 (0)	8 (2.7)	
Secondary (fifth to seventh)	54 (18.0)	64 (21.3)	
High school(eighth to tenth)	148 (49.3)	152 (50.7)	
Pre-degree	70 (23.3)	50 (16.7)	
Graduate	24 (8.0)	24 (8.0)	
Postgraduate	4 (1.3)	0 (0)	
Occupation			8.123, 0.017*
Housewife	286 (95.3)	298 (99.3)	
Manual laborer	0 (0)	2 (0.7)	
Office worker	14 (4.7)	0 (0)	
Family Income (monthly, Indian Rs)	204	204	6.87, 0.076
1500 – 3000	(68.0)	(68.0)	
3001 – 4500	58 (19.3)	76 (25.3)	
4501 – 6000	28 (9.3)	20 (6.7)	
≥ 6001	10 (3.3)	0 (0)	
Type of family			2.06, 0.356
Nuclear	78 (26.0)	70 (23.3)	
Three generation	100 (33.3)	124 (41.3)	
Joint	122 (40.7)	106 (35.3)	
Gravida			1.17,0.557
Primi	132 (44.0)	118 (39.3)	
Second Gravida	112 (37.3)	112 (37.3)	
≥ Third Gravida	56 (18.7)	70 (23.3)	

* *P* value less than 0.05 is considered as significant

Body mass index ≥ 25 was significantly higher in cases than controls (37.9 vs. 14.3%). Around 24% of the cases and 11.3% of the controls had a history of irregular menstrual cycle. The proportion of subjects taking treatment for infertility was high among the cases (18.7%) as compared to controls (5.3%). Similarly, a proportion of those with a family history of diabetes among first-degree relatives and especially in the mother were more among cases as compared to controls, and the differences found in all the above-mentioned factors were statistically significant. Incidence of diabetes in fathers of women with GDM was 11.33%, while in controls it was 5.33% (*P* = 0.093). The incidence of diabetes in mothers was 21.33% in cases vs. 8.33% in controls (*P* = 0.003) [Table 2].

**Table 2:** Determinants for GDM according to personal and family history

Determinants	Cases (%)	Control (%)	OR (95% CI)	χ^2^, *P*
Body Mass Index				
< 25	154 (62.1)	168 (85.7)	3.7 (2.3-5.9)	15.322, <0.001*
≥ 25	94 (37.9)	28 (14.3)		
Menstrual cycle				
Regular	228 (76)	266 (88.7)	2.5 (1.6-3.9)	8.273, 0.006*
Irregular	72 (24)	34 (11.3)		
Treated for infertility				
Yes	56 (18.7)	16 (5.3)	4.1 (2.3-7.3)	12.626, 0.001*
No	244 (81.3)	284 (94.7)		
Family h/o diabetes in first-degree relatives				
Yes	112 (37.3)	36 (12.0)	4.4 (2.9-6.6)	25.903, 0.001*
No	188 (62.7)	264 (88.0)		
H/o diabetes in mother				
Yes	32 (21.3)	13 (8.7)	2.9 (1.4-5.7)	8.41, 0.003*
No	118 (78.7)	137 (91.3)		
h/o Diabetes in father				
Present	17 (11.3)	8 (5.3)	2.3 (0.9-5.4)	2.79, 0.093
Absent	133 (88.7)	142 (94.7)		

* *P* value less than 0.05 is considered as significant

Univariate analysis also revealed that history of previous pregnancy losses (OR = 2.4), past GDM (OR = 5.3), pre-maturity (OR = 10.6), pre-eclampsia (OR = 1.8), UTI (OR = 4.8), and moniliasis (OR = 11.8); polyhydramnios (OR = 6.9), macrosomia (OR = 4.4), and pre-term labor (OR=2.6) were significantly associated with the presence of GDM. About 68.96% of the women with previous losses had GDM as against 31.03% of the controls [Tables 3 and 4].

**Table 3:** Univariate analysis showing the determinants for GDM according to past history

Determinants	Cases (%)	Control (%)	OR (95% CI)	χ^2^, *P*
H/o abortion				
Present	236 (78.7)	242	0.9 (0.6-1.3)	0.185, 0.774
Absent	64 (21.3)	(80.7) 58 (19.3)		
H/o Previous fetal losses				
Yes	40 (13.3)	18 (6.0)		
No	260 (86.7)	282 (94.0)	2.4 (1.3-4.3)	4.619, 0.049*
H/o congenital fetal anomalies				
Present	6 (2.0)	2 (0.7)	3.0 (0.6-15.2)	1.014, 0.622
Absent	294 (98.0)	298 (99.3)		
H/o fetal macrosomia				
Present	8 (2.7)	2 (0.7)	4.1 (0.9-19.4)	1.831, 0.371
Absent	292 (97.3)	298 (99.3)		
H/o past GDM				
Present	20 (6.7)	4 (1.3)	5.3 (1.8-15.7)	5.556, 0.035*
Absent	280 (93.3)	296 (98.7)		
H/o Hydramnios				
Present	6 (2.0)	4 (1.3)	1.5 (0.4-5.4)	0.203, 0.989
Absent	294 (98.0)	296 (98.7)		
H/o prematurity				
Present	20 (6.7)	2 (0.7)	10.6 (2.5-46.0)	7.644, 0.01*
Absent	280 (93.3)	298 (99.3)		

*P* value less than 0.05 is considered as significant

**Table 4:** Univariate analysis showing the determinants for GDM according to present history

Determinants	Cases (%)	Control (%)	OR (95% CI)	χ^2^, *P*
H/o pre-eclampsia				
Present	88 (29.3)	56 (18.7)	1.8 (1.2-2.7)	4.678, 0.04*
Absent	212 (70.7)	244 (81.3)		
H/o UTI				
Present	110 (36.7)	32 (10.7)	4.8 (3.1-7.5)	28.064, <0.001*
Absent	190 (63.3)	268 (89.3)		
H/o moniliasis				
Present	112 (37.3)	16 (5.3)	11.8 (6.8-20.7)	45.76, <0.001*
Absent	168 (62.7)	284 (94.7)		
Liquor volume				
Normal	208 (68.7)	262 (87.3)	-	18.31, <0.001*
Increased	44 (14.7)	8 (2.7)	6.9 (3.2-15.0)	
Macrosomia				
Present	46 (15.3)	6 (2)4.4	(1.0-19.1)	16.844, <0.001*
Absent	254 (84.7)	294 (98)		
H/o Preterm labor				
Present	34 (11.3)	14 (4.7)	2.6 (1.4-5.0)	4.529, 0.05*
Absent	266 (88.7)	286 (95.3)		

*P* value less than 0.05 is considered as significant

Multivariate logistic regression analysis identified the following significant determinants: pre-pregnancy BMI of ≥ 25 (OR = 2.7), treatment for infertility (OR = 3.3), family history of diabetes (OR = 4.5), history of UTI (OR = 3.2), history of moniliasis (OR = 7.6), polyhydramnios (OR = 6.0), macrosomia (OR = 4) [Table 5].

**Table 5:** Multivariate logistic regression analysis

Determinants	*P* value	Adjusted OR	95% C.I. for OR	
			
			Lower	Upper
BMI				
< 25	0.017*	2.695	0.164	8.835
≥ 25				
Treatment for infertility				
Yes	0.029*	3.281	1.127	9.547
No				
Family History of diabetes				
Yes	< 0.001*	4.542	2.041	10.106
No				
H/o UTI				
Yes	0.005*	3.225	1.412	7.365
No				
H/o Moniliasis				
Yes	< 0.001*	7.583	2.886	19.921
No				
Liquor volume				
Normal	0.018*	5.964	1.353	26.279
Increased				
Macrosomia				
Yes	0.049*	4.389	1.008	19.117
No				

* *P* value less than 0.05 is considered as significant, Method = Forward Stepwise (Likelihood Ratio)

## Discussion

This study provides baseline information about the determinants of GDM, which could potentially help to incorporate early intervention measures. There was an increase in the frequency of gestational diabetes among women who had a history of infertility as illustrated in a study in the Indian Diabetic Clinic.[3] Irregular menstrual cycle was also found to be more in cases who developed GDM.[4,5] Our study showed that overweight and obese women were more prone to develop GDM, as observed in other studies.[3,6–8] Increased BMI and insulin resistance is also linked to polycystic ovary syndrome (PCOS), especially in Indian subcontinent Asian women. A study in Iran (May, 2008) and studies in other countries came to a conclusion that women with PCOS had a higher risk of developing GDM.[9–11] Another study in Bangkok, Thailand also came to the same conclusion that prevalence of GDM in Asian women with PCOS was very high.[12] Thus obesity, which is linked to PCOS, infertility, and irregular menstrual history were found to be important risk factors in our study.

A prospective case control study in China in 2005, reported that a family history of diabetes greatly increased the incidence of GDM.[13] Similar results were reported by other studies.[13–15] Another study compared the prevalence of maternal and paternal history of diabetes in the proband with GDM and the analysis did not show any statistical significance.[16] In 2000, in a population-based study, it was reported that the history of diabetes in the patient's mother was significantly associated with a risk of GDM, besides, subsequently developing T2DM later on in life.[17] The maternal, but not paternal, association suggested that although a familial tendency definitely exists, this was probably not a purely genetic influence. The familial association was most probably the product of the minor alterations that occurred in the intrauterine ‘milieu interieur’ of the infant in the mother, with abnormal carbohydrate metabolism. This has been previously suggested by epidemiological studies in other populations, notably the Pima Indians[18] and in an animal model.[19] This shows that there is a highly significant risk with maternal association as against paternal association toward the development of GDM.

According to a study (Canada, in December, 2001), one of the independent risk factors for development of GDM was previous unexplained neonatal deaths.[20] A population-based longitudinal study concluded that mild pre-eclampsia and chronic hypertension, with superimposed pre-eclampsia, occurred more frequently in women with GDM.[21,22] In contrast to this, another study in June 2007, came to the conclusion that prevalence of pre-eclampsia was not increased in women presenting with GDM.[23] Our study has highlighted that history of previous losses and pre-eclampsia are associated with GDM.

The history of macrosomia in previous pregnancy was not found to be a risk factor for GDM, similar to another study.[15] The present study showed that women with a history of GDM in a previous pregnancy were more likely to have GDM in the present pregnancy, reflecting the inherent tendency of women to develop insulin insensitivity. A study reported that in patients with a history of GDM, the risk for recurrence increased if GDM was diagnosed earlier, as they required insulin, had elevated third-trimester plasma glucose level, and delivered macrosomic infants in their index pregnancy.[24] In a longitudinal study conducted in USA in 1998, it was reported that urinary tract infection occurred more frequently in women with GDM than in those in whom diagnosis was not made.[21] A case-control study in China also published the same results, with regard to moniliasis.[13] A past history of GDM and infections such as UTI and moniliasis, are the other associated factors in our study.

To summarize, pre-pregnancy overweight and obesity, irregular menstrual cycle, history of treatment for infertility, family history of diabetes in first-degree relatives, history of diabetes in mother, history of previous pregnancy losses, past GDM, pre-maturity, pre-eclampsia, UTI, moniliasis, polyhydramnios, macrosomia, and pre-term labor were significantly associated with the presence of GDM in a univariate analysis. Pre-pregnancy BMI of ≥ 25, treatment for infertility, family history of diabetes, history of UTI, history of moniliasis, polyhydramnios, and macrosomia were independently associated with GDM as shown by multiple logistic regression analysis. In view of the above-mentioned findings, it is concluded that GDM is associated with several different modifiable and non-modifiable risk factors in our study.

As this was a hospital-based case control study, it could have been biased to a certain extent. Although increased parity is a known risk factor for GDM, it does not come as significant in our study. This might be due to the fact that hospital controls are often a source of selection bias. Besides, the confounding effect of some other unknown factors may have a role to play. In spite of these constraints, the study provides valuable information, which can be helpful in planning maternal health services, by early identification and providing high quality prenatal care to GDM women. Also screening of risk-pregnant patients will be cost-effective, especially in a developing country. We recommend that health authorities strengthen maternal health programs by focusing on the prevention and control of modifiable risk factors during the pre-pregnancy period and introducing corrective therapeutic interventions such as exercise and dietary modification.
